# Virtual Screening of Natural Chemical Databases to Search for Potential ACE2 Inhibitors

**DOI:** 10.3390/molecules27051740

**Published:** 2022-03-07

**Authors:** Huiping Yao

**Affiliations:** Department of Obstetrics and Gynecology, The First Hospital of Lanzhou University, Lanzhou 730013, China; ldyy_huipingyao@lzu.edu.cn

**Keywords:** ACE2, inhibitor, natural products, virtual screening, molecular dynamics simulation

## Abstract

The angiotensin-converting enzyme II (ACE2) is a multifunctional protein in both health and disease conditions, which serves as a counterregulatory component of RAS function in a cardioprotective role. ACE2 modulation may also have relevance to ovarian cancer, diabetes, acute lung injury, fibrotic diseases, etc. Furthermore, since the outbreak of the coronavirus disease in 2019 (COVID-19), ACE2 has been recognized as the host receptor of severe acute respiratory syndrome coronavirus 2 (SARS-CoV-2). The receptor binding domain of the SARS-CoV-2 S-protein has a strong interaction with ACE2, so ACE2 may be a potent drug target to prevent the virus from invading host cells for anti-COVID-19 drug discovery. In this study, structure- and property-based virtual screening methods were combined to filter natural product databases from ChemDiv, TargetMol, and InterBioScreen to find potential ACE2 inhibitors. The binding affinity between protein and ligands was predicted using both Glide SP and XP scoring functions and the MM-GBSA method. ADME properties were also calculated to evaluate chemical drug-likeness. Then, molecular dynamics (MD) simulations were performed to further explore the binding modes between the highest-potential compounds and ACE2. Results showed that the compounds 154-23-4 and STOCK1N-07141 possess potential ACE2 inhibition activities and deserve further study.

## 1. Introduction

Angiotensin-converting enzyme (ACE) is a highly glycosylated transmembrane protein existing in two differentially spliced forms: the two-domain somatic ACE (ACE1, N- and C-domains) with similar, though not identical, substrate specificities and the single-domain testicular form (ACE2). Donoghue et al. identified the ACE2 gene as one that was upregulated in a human heart failure cDNA library [1]. ACE2 actually serves a multiplicity of functions and plays vital roles in different diseases, such as ovarian cancer, diabetes, acute lung injury, fibrotic diseases, etc. [2]. ACE2 is very common in ovarian cancer with amplification mutations. High expression of ACE2 promotes the prognosis of patients with ovarian cancer [3]. Recently, it has been proven that human ACE2 is a host receptor of severe acute respiratory syndrome coronavirus (SARS-CoV), which could specifically bind to SARS-CoV spike protein with high affinity [4,5]. A newly published paper reported that the novel coronavirus could enter ACE2-expressing cells, but not the cells that did not express ACE2, so ACE2 is also the host receptor for severe acute respiratory syndrome coronavirus 2 (SARS-CoV-2) [6].

SARS-CoV-2 is a well-known novel coronavirus that causes an acute infectious pneumonia disease, coronavirus disease 2019 (COVID-19) [7,8]. Symptoms of the infection include respiratory symptoms, fever, cough, shortness of breath, and breathing difficulties. In more severe cases, infections can cause pneumonia, severe acute respiratory syndrome, kidney failure, and even death [9,10,11]. It has been reported that bats might be the original host of this virus [12,13,14] and some animals sold at seafood markets may be the intermediate hosts for novel coronavirus [15]. The World Health Organization (WHO) announced that the novel coronavirus epidemic was listed as a Public Health Emergency of International Concern (PHEIC) on 30 January 2020. According to the COVID-19 Dashboard by the Center for Systems Science and Engineering at Johns Hopkins University (https://www.arcgis.com/apps/opsdashboard/index.html#/bda7594740fd40299423467b48e9ecf6 accessed on 30 October 2021), up to 1 October 2021, COVID-19 had caused more than 233 million confirmed cases and 4.7 million deaths across 192 countries and areas.

The common structure of coronavirus consists of spike (S), envelope (E), membrane (M), and nucleoprotein (N) [16,17,18,19]. The genus of coronavirus has been divided into four genera: α-coronavirus, β-coronavirus, γ-coronavirus, and δ-coronavirus [13,20]. As with SARS-CoV, the SARS-CoV-2 sequence also belongs to β-coronavirus [21]. The sequence similarities reach 76.04%, 73.33%, and 50.00% for the whole protein, receptor-binding domain (RBD), and receptor-binding motif (RBM) between the SARS-CoV-2 spike and the SARS-CoV spike (isolated from human), respectively [22]. Receptor recognition is the first essential step in the viral infection of host cells, and spike protein has been reported to mediate the entry of the virus into host cells [23,24,25]. Paxlovid was authorized by the FDA as the first oral antiviral method for the treatment of COVID-19 on 22 December 2021. Paxlovid is a mixture of nirmatrelvir and ritonavir, which can be used to treat mild to moderate COVID-19 adult and child patients (12 years and older and at least 40 kg) [26]. Therefore, it is extremely urgent to discover new drugs to inhibit novel coronavirus as soon as possible.

The search for an ACE2 inhibitor or activator could promote drug discovery for several diseases. Specifically, the inhibition of ACE2 may prevent the invasion of SARS-CoV-2. In this study, we carried out a combined virtual screening protocol to search for potential ACE2 inhibitors. Molecular docking was used to screen three natural compound product databases (ChemDiv, TargetMol, and InterBioScreen) targeting ACE2. The molecular mechanics-generalized Born surface (MM-GBSA) was also calculated to evaluate the binding of chemicals to ACE2. The ADME properties were used to measure chemical drug-likeness. Clustering analysis based solely on the structural information was performed to aid the selection of potential ACE2 inhibitors with various skeletons. Molecular dynamics simulation was used to investigate the binding mode of the inhibitors with ACE2.

## 2. Results

### 2.1. Molecular Docking Screening

**Figure 1:**
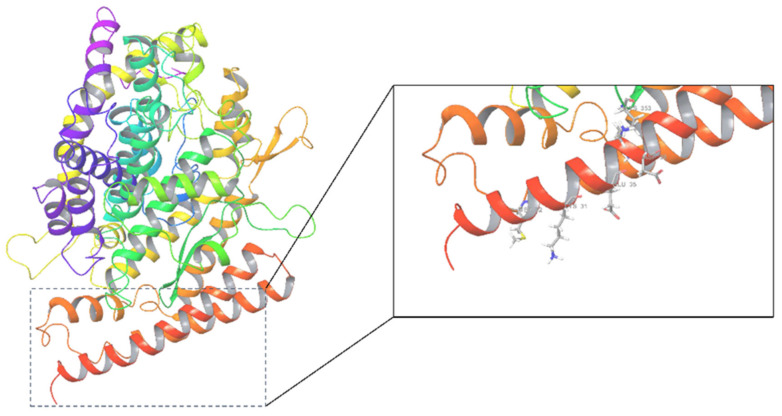
The crystal structure of ACE2 (1R42) and its active center.

The structures of the human angiotensin-converting enzyme ACE2 (PDB ID: 1R42) and the ligand binding sites are shown in Figure 1. A total of 70,902 natural compounds were combined as a ligand database, including 398 compounds from pure natural products of ChemDiv, 2131 compounds from pure natural products of TargetMol, and 68,373 compounds from the InterBioScreen natural subset. These chemicals were neatened, minimized, and prepared for docking screening to predict their binding affinities and molecular recognition using Glide SP (standard precision), with an output 42,614 molecules. These compounds had SP Glide scores ranging from −0.023 to −7.626 kcal/mol. Higher negative docking score values indicate higher affinity between the receptor and the ligands [27].

Glide XP (extra precision) is a docking method superior to SP docking [28]. A total of 10,451 out of 42,614 molecules (about the top-ranked 25%) were subjected to Glide XP calculation, and 9678 molecules were successfully docked to the receptor with Glide scores ranging from 2.951 to −7.997.

### 2.2. MM-GBSA

Then, the 500 top-ranked complexes of the XP docking score were chosen to calculate the MM-GBSA values (ΔG), which were used to assess the binding abilities of the receptor and ligands [29]. The obtained MM-GBSA ΔG values ranged from 0.839 to −60.737 kcal/mol.

### 2.3. ADME Analysis

ADME properties were calculated for the 500 top-ranked ligands after MM-GBSA treatment. The criteria contained in the rule of five were: (I) molecular mass less than 500 dalton, (II) partition coefficient (QPlogPo/w) not greater than 5, (III) hydrogen bond donors less than 5, (IV) hydrogen bond acceptors less than 10, (V) PSA less than 140 Å^2^, and (VI) percent human oral absorption more than 25. These rules were selected to evaluate the drug-likeness of compounds or to determine if a compound had pharmacological or biological potency [30]. A total of 298 ligands remained after deleting molecules that did not meet these standards.

### 2.4. Cluster Analysis

Cluster analysis was executed based on the molecular structural information. The remaining 298 molecules were clustered into 12 different categories, and the compound number contained in each category is shown in Table 1. The chemical with the best docking score in every category was chosen for further analysis.

### 2.5. Virtual Screening Results

**Figure 2:**
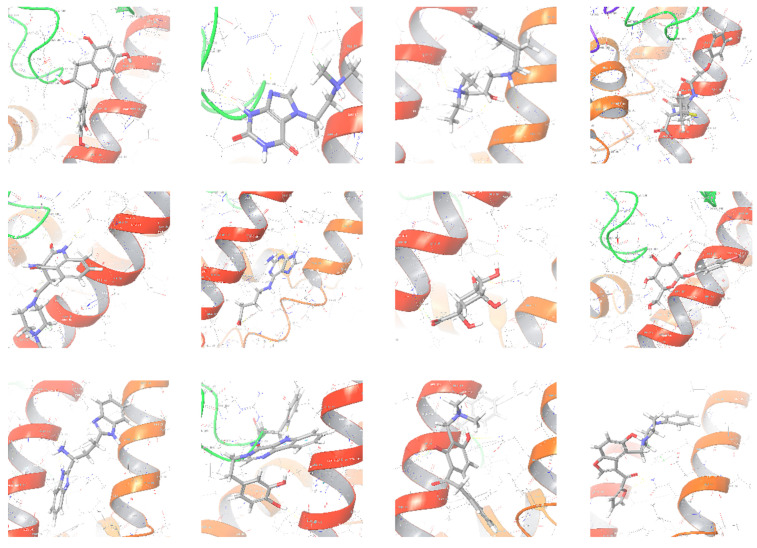
The interactions between the receptor and the 12 top ligands. The yellow line shows a hydrogen bond, the blue lines show π-π interactions, and the green lines show salt bridges.

The selected representative structures of the 12 ligands with their corresponding ADME properties are shown in Table 2. The interactions between the receptor and top 12 ligands are shown in Figure 2. Their docking results and binding residues are listed in Table 3 (The figures shown in this table were depicted using Maestro 10.1 (Schrödinger Inc., LLC, New York, NY, USA)). The structures of the 12 ligands are mostly phenol, ketone, and amine compounds.

**Figure 3:**
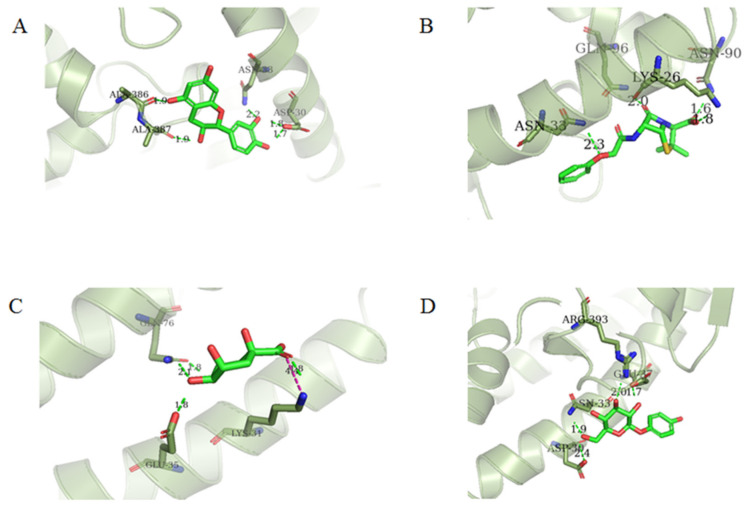
The interaction details of four potential compounds with ACE2. (**A**) 154-23-4; (**B**) 132-98-9; (**C**) STOCK1N−53429; and (**D**) STOCK1N-07141. The green lines show hydrogen bonds, and the purple line shows salt bridges.

Four of the twelve ligands were found to have clear sources and medicinal effects as known drug ingredients; these were compounds 154-23-4, 132-98-9, STOCK1N−53429, and STOCK1N-07141. With the increase in confirmed COVID-19 cases and deaths, it is a good strategy to find novel effects from known drugs or ingredients to prevent the invasion of SARS-CoV-2 because drug repositioning possesses several advantages when considering time, research cost, and safety [31]. Therefore, compounds 154-23-4, 132-98-9, STOCK1N−53429, and STOCK1N-07141 were selected for further analysis, and their interactions and distance are shown in Figure 3 (figures were depicted using PyMOL Molecular Graphics System Version 2.5.2 (Schrödinger, Inc., LLC)).

The first natural product was 154-23-4 (catechin), a polyphenolic compound found in the bark and twigs of plants [32], which possesses antioxidant, anti-inflammatory, antibacterial, antifungal, antiviral, and anticancer properties [33,34,35,36]. Catechin also possesses virus inhibition activity, and the EC50 for the influenza A (H1N1) virus is 18.4 μg/mL [36]. The Glide docking results showed strong interaction between catechin and ACE2. The three OH from chromane in 154-23-4 formed an OH-O hydrogen bonding interaction with the backbone carbonyl atom from Ala387 with a distance of 1.9 Å. The five OH from chromane formed an OH-O interaction with the backbone carbonyl atom from Ala386 with a distance of 1.9 Å. The three OH from phenyl in 154-23-4 formed an OH-N interaction with the amide -NH from Asn33 with a distance of 2.2 Å and formed an OH-O interaction with the oxygen from carboxyl in Asp30 with a distance of 1.8 Å. The four OH from phenyl formed an OH-O interaction with the oxygen from carboxyl in Asp30 with a distance of 1.7 Å. All these results indicated a strong interaction between the ligand and protein with a Glide score of −5.418 and a value of −37.592 kcal/mol.

The second natural product was 132-98-9 (Penicillin V Potassium), which is useful for the treatment of bacterial infections [37]. The docking results indicated that Penicillin V Potassium could bind to ACE2 very well. The oxygen from carboxyl in 132-98-9 formed an O-HN interaction with the -NH from Lys26 with a distance of 1.8 Å. The carbonyl from carboxyl formed an O-HN interaction with the -NH from Asn90 with a distance 1.6 Å. The carbonyl from β-lactam in 132-98-9 formed an O-HN interaction with the -NH from Gln96 with a distance 2.0 Å. The oxygen from phenoxy in 132-98-9 formed an O-HN interaction with the -NH from Asn33 with a distance of 2.3 Å. The Glide score and ΔG value were −4.335 and −18.899 kcal/mol, respectively.

The third natural compound was STOCK1N−53429 (quinic acid), a widely presented natural product found in plants [38] Quinic acid has antioxidants, increases urinary excretion, and enhances DNA repair and immunity properties [39,40,41,42]. Glide docking results showed good affinity between quinic acid and ACE2 with four hydrogen bonding interactions and a salt-bridge interaction. The three OH from cyclohexane in STOCK1N−53429 formed an OH-O interaction with amide carbonyl from Gln76 with a distance of 1.8 Å. The OH from cyclohexane formed an O-HN interaction with the -NH from Gln76 with a distance of 2.1 Å, as well as an OH-O interaction with oxygen from carboxyl in Glu35 with a distance of 1.8Å. Carbonyl in STOCK1N−53429 formed an O-HN interaction with the -NH from Lys31 with a distance of 1.8 Å. Furthermore, there was a salt-bridge interaction between STOCK1N−53429 and Lys 31 with a distance of 4.4 Å. The Glide score and ΔG value were −5.923 and −19.312 kcal/mol, respectively.

The fourth natural compound was STOCK1N-07141 (arbutin), which was first discovered in the leaves of the bearberry plant and is widely found in animals, plants, and microbes [43,44]. Arbutin has been shown to have antioxidant, anti-inflammatory, and antibacterial properties [45,46,47]. Hydroxymethyl in STOCK1N-07141 formed an OH-O interaction with the oxygen from carboxyl in Asp30 with a distance of 2.4 Å and formed an O-HN interaction with the -NH from Asn33 with a distance of 1.9 Å. The two OH in STOCK1N-07141 formed an O-HN interaction with the -NH from Arg393 with a distance of 2.0 Å and formed an OH-O interaction with the oxygen from carboxyl in Glu37 with a distance of 1.7 Å. The Glide score was −4.898, and the ΔG value was −27.518 kcal/mol.

These four chemicals have been found to possess medicinal effects, including enhanced immunity, anti-inflammatory, and antibacterial properties, etc. In addition, chemical 154-23-4 has antiviral effects. From the screening results, we knew that chemicals 154-23-4 and STOCK1N−53429 both had five interactions, including hydrogen and salt-bridge, with ACE2, and ligands 132-98-9 and STOCK1N-07141 had four hydrogen interactions with ACE2. These four compounds all had strong binding abilities with ACE2. In order to better determine the binding stability and binding modes of these four compounds and ACE2, we performed MD simulations on the systems combining these four compounds with ACE2.

### 2.6. Molecular Dynamics Simulation Results

**Figure 4:**
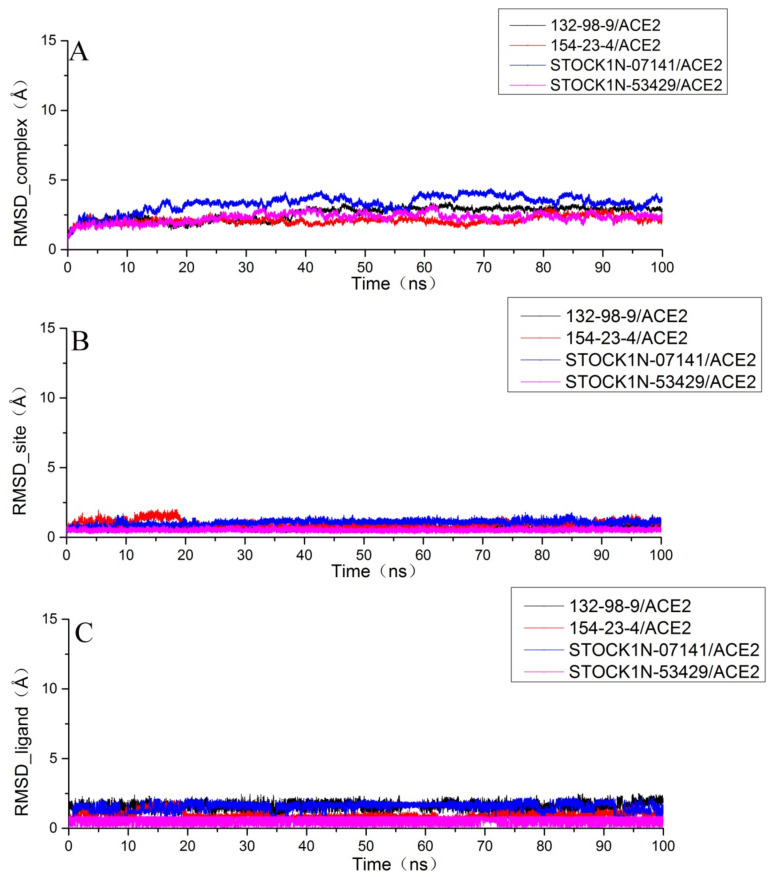
Root mean square deviation (RMSD) of each system. (**A**) The RMSD of the protein backbone atoms of the complex; (**B**) the RMSD of the heavy atoms (all non-hydrogen atoms) of the ligand; and (**C**) the RMSD of the Cα atoms at the binding site with residues within 5 Å around the ligand.

After running a 100 ns molecular dynamics simulation for each system, we analyzed the obtained trajectory of each system. In order to research the stability of the structure, we calculated the root mean square deviation (RMSD) of the complex, the ligand, and the binding pocket (defined as residues within 5 Å around the ligand). As shown in Figure 4A, each system had a certain fluctuation in the initial stage of the molecular dynamics simulation and then gradually stabilized in the last 20 ns. In order to obtain more accurate analytical results, we chose the last 20 ns trajectory of each system for the succeeding analysis.

**Figure 5:**
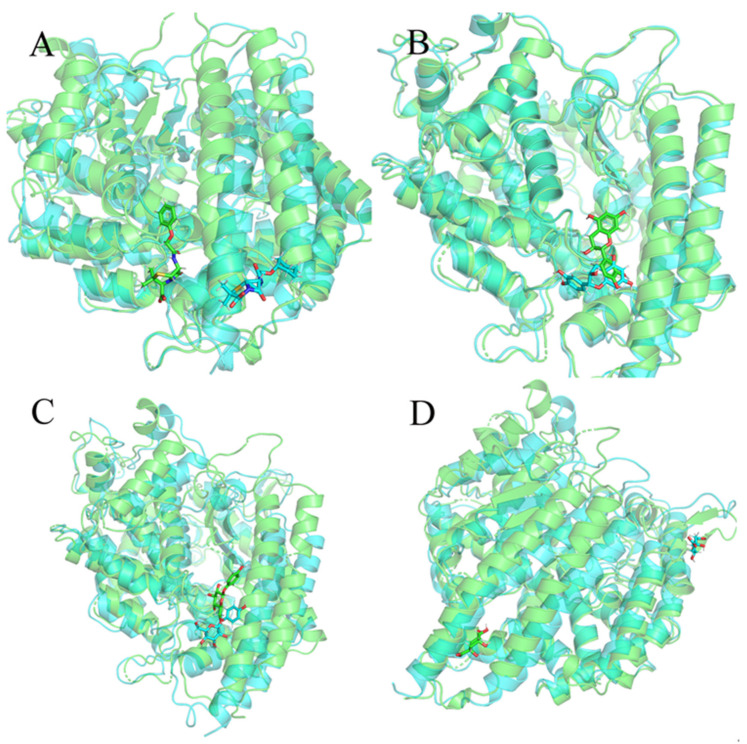
Overlay of the last frame structure of the trajectory and the initial structure. Green is the initial structure, and blue is the last frame structure from the trajectory. (**A**) 132-98-9/ACE2, (**B**) 154-23-4/ACE2, (**C**) STOCK1N-07141/ACE2, and (**D**) STOCK1N−53429/ACE2.

Then, we extracted the last frame structure from the trajectory of each system and superimposed them with the initial structures to determine whether the small molecule was still at the binding site after the molecular dynamics simulation. As shown in Figure 5A, we found that the binding position of 132-98-9 in ACE2 changed. As shown in Figure 5B,C, although the conformation of 154-23-4 and STOCK1N-07141 underwent some changes, they remained stable at the binding site. As shown in Figure 5D, STOCK1N−53429 could not stably exist at the binding site. Since STOCK1N−53429 could not bind to ACE2 stably, we no longer considered this small molecule in subsequent analyses.

**Figure 6:**
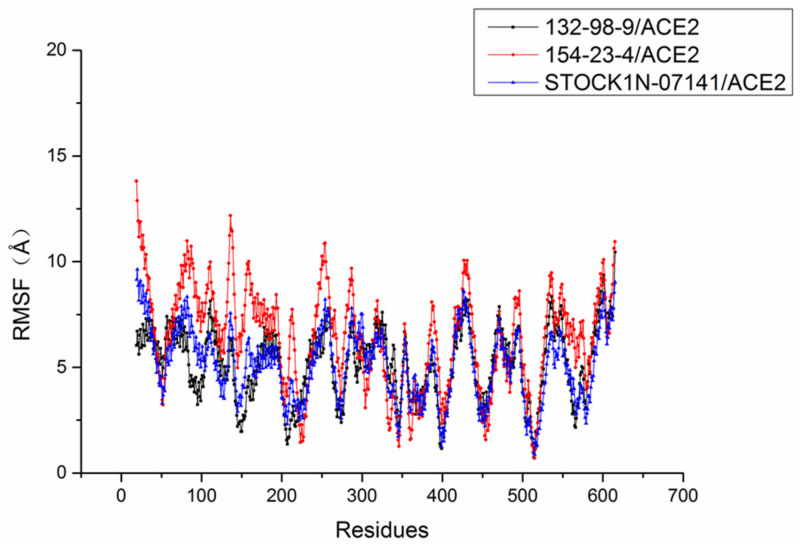
Root mean square fluctuation (RMSF) of each system.

The root mean square fluctuation (RMSF) of each residue was calculated to research the stability of residues in these systems. As shown in Figure 6, the RMSF trends of these three systems were very similar. Compared with the stability of amino acid residues in other systems, the residues in the 154-23-4/ACE2 system had higher RMSF values, which indicated that the residues in the 154-23-4/ACE2 system were more unstable than other systems during the molecular dynamics simulation.

The binding energy between the ligand and protein of each system was calculated using the MM-GBSA method and is listed in Table 4. From the values of binding free energy (∆G_bind_), we concluded that these three compounds bind well to ACE2. At the same time, we found that the contribution of electrostatic interaction energy (∆E_ele_) was quite different in the three systems. The contribution of electrostatic interaction energy presented a higher positive value in the 132-98-9/ACE2 system, while it presented a negative value in both the 154-23-4/ACE2 system and the STOCK1N-07141/ACE2 system. This indicated that electrostatic interaction energy was not conducive to the binding of ligand and protein in the 132-98-9/ACE2 system, but it had a positive effect on the binding of ligand and protein in both the 154-23-4/ACE2 system and the STOCK1N-07141/ACE2 system.

Meanwhile, we found that the contribution of polar solvation free energy (∆G_GB_) was also quite different in the three systems. The value of polar solvation free energy was negative in the 132-98-9/ACE2 system, but it was opposite in the other two systems. This result showed that polar solvation free energy was beneficial to the binding of ligand and receptor in the 132-98-9/ACE2 system, while it had a disadvantageous effect on the binding of ligand and protein in the other two systems. The difference in the contributions of electrostatic interaction energy and polar solvation free energy to the three systems may be due to the difference in binding sites.

**Figure 7:**
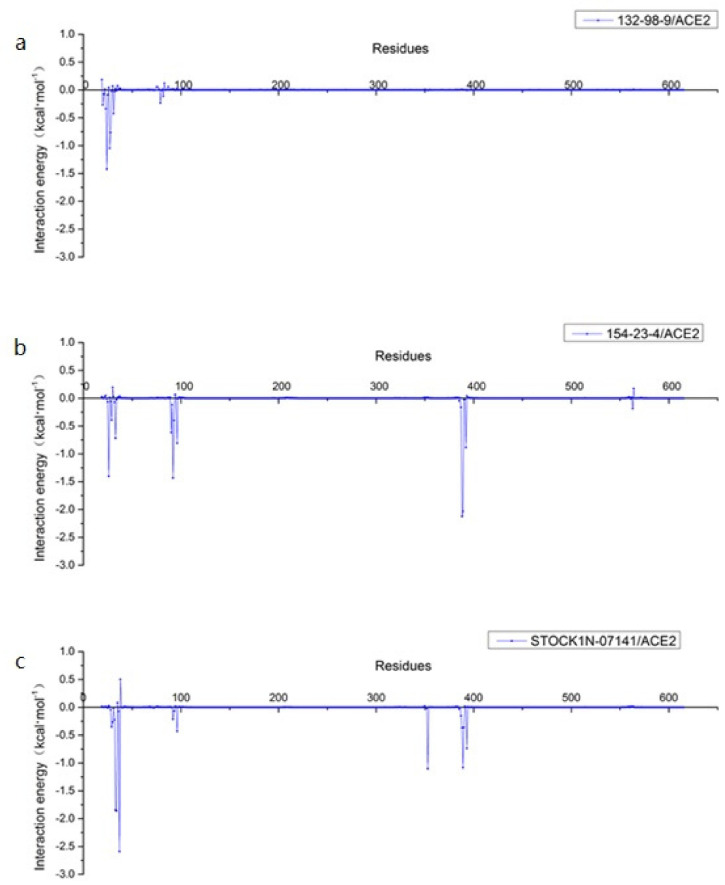
Contributions of residues calculated by decomposing the binding energy. (**a**) 132-98-9/ACE2, (**b**) 154-23-4/ACE2, and (**c**) STOCK1N-07141/ACE2.

**Figure 8:**
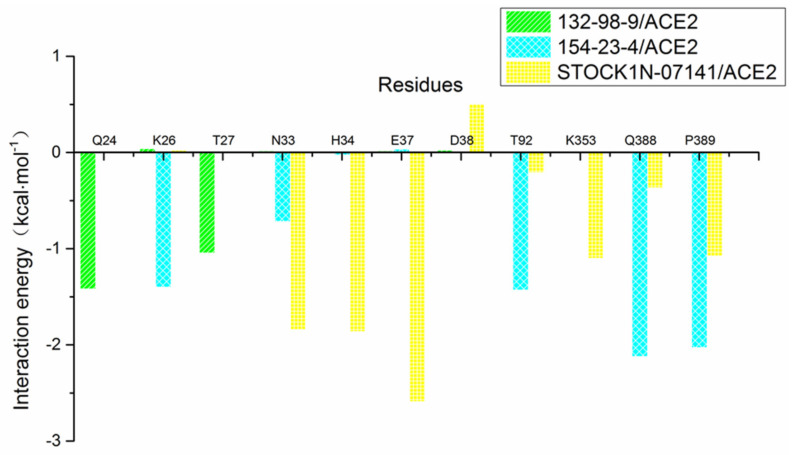
Contributions of partial residues calculated by decomposing the binding energy.

In addition, we decomposed the binding energy to study the contribution of each residue, as shown in Figure 7. In this figure, the key amino acid residues presenting high energy contributions were different in each of the three systems. In order to more clearly observe the energy contributions of these amino acid residues in the different systems, we extracted and mapped the energy contributions of these amino acid residues. As shown in Figure 8, Q24 and T27 in the 132-98-9/ACE2 system presented a higher contribution to the binding, and they showed almost no energy contribution in the other two systems. This situation may be related to the changed binding site in the 132-98-9/ACE2 system. At the same time, although the binding sites of the other two systems did not change, the energy contributions of their amino acid residues still showed large differences. K26, T92, Q388, and P389 had high energy contributions in the 154-23-4/ACE2 system, while N33, H34, E37, and K353 had high energy contributions in the STOCK1N-07141/ACE2 system.

In order to explore the reasons why the energy contributions of amino acid residues were different in the 154-23-4/ACE2 system and the STOCK1N-07141/ACE2 system, we calculated the hydrogen bonds (defined as the distance between acceptor and donor <0.35 nm and an angle >120°) of each system during the MD simulation. As shown in Table 5, hydrogen bond interaction had little effect on the 132-98-9/ACE2 system, while Q388 formed a hydrogen bond with the ligand in the 154-23-4/ACE2 system, which may be the reason for its greater contribution in this system. In the MD process, the hydrogen bond between E37 and the ligand occupied a higher proportion. This could be the reason why E37 contributed the most to the integration of the entire system. The difference in hydrogen bonds may be the reason for the differences in the energy contributions of the residues in different systems.

## 3. Materials and Methods

### 3.1. Protein Preparation

The crystal structure of ACE2 (PDB ID: 1R42) was obtained from the Protein Data Bank and prepared using the Protein Preparation Wizard in Maestro. After deleting water, adding hydrogens, and filling in missing side chains, the protein was optimized at PROPKA pH, and the side chains were minimized with a liquid simulation OPLS_2005 force field. The grid was generated using receptor grid generation protocol. As Wan et al. reported, residues 31, 35, 38, 82, and 353 play a critical role in the affinity between ACE2 and SARS-CoV-2 [22]. These residues were chosen as active center, and the receptor grid size was defined as a 15 Å box. Other adjustable settings were set to default.

### 3.2. Ligand Preparation

Natural product databases from ChemDiv (398), TargetMol (2131), and InterBioScreen (68373) were combined for virtual screening. All molecular structures with ionization states were generated at a specific pH of 7.0 ± 2.0 [28], and stereoisomer computation was left at determined chiralities using the Lig Prep protocol on an OPLS_2005 force field [48].

### 3.3. Molecular Docking

Molecular docking was implemented using a glide docking package. The prepared ligands were docked into the active site of the protein using standard precision (SP), which considers the ligands as rigid, followed by extra precision (XP), which considers the ligands as flexible. Glide SP is a softer docking method that seeks to minimize false negatives; Glide XP is a harder function that exacts severe penalties for poses that violate established physical chemistry [49]. The docked conformers were evaluated using a docking score.

The binding free energies of the docked receptor and ligand complexes were calculated using the prime molecular mechanics-generalized Born surface (MM-GBSA) protocol on an OPLS_2005 force field [50]. 

### 3.4. ADME Analysis

Absorption, distribution, metabolism, and excretion (ADME) properties are crucial to the development of new drugs [51]. ADME properties were calculated to screen higher quality molecules and identify whether the molecules have drug-formability [52]. The Quik Prop protocol was employed to predict the molecular ADME properties and evaluate the drug-like properties stated in the rule of five (RO5) [53]. 

### 3.5. Cluster Analysis

The obtained ligands after docking and ADME screening were selected to export data files (cnv format) for clustering using Cavas’ tool to obtain different molecular scaffolds. The representative compounds with the highest XP Glide scores among the different clusters were selected for further analysis.

### 3.6. Molecular Dynamics Simulation

In order to further study these systems, AMBER14 [54]. was used to run molecular dynamics simulations (MD). We used Gaussian 09 at the HF/6-31G* level of theory to optimize the ligands, and we used the restrained electrostatic potential (RESP) protocol to calculate the partial atomic charges of the ligand atoms [55,56,57]. Then, the general AMBER force field (GAFF) [58]. was used to create force field parameters for the ligands, and the standard ff14SB [59]. force field was used to generate force field parameters for the protein. Then, the tLEaP module of AMBER14 was executed to add the hydrogen atoms and the appropriate number of Na+ to the systems. Then, we wrapped each system with a TIP3P cube box [60]. with each atom in the system at least 10 Å from the edge of the water box. Then, the Sander program was used to operate the minimization, heating, density, and equilibration protocols.

In this study, we performed a three-step minimization procedure with a limiting force of 5.0 kcal·mol^−1^·Å^−2^ on all atoms, a limiting force of 3.0 kcal·mol^−1^·Å^−2^ on the protein backbone atoms, and no restriction on all atoms. We ran 5000 steps for each minimization: the steepest descent method was used for the first 2500 steps, and the conjugated gradient method was used for the last 2500 steps. After minimization, each system was heated from 0.0 K to 310.0 K in an NVT ensemble. In this step, we imposed a limit force of 5.0 kcal·mol^−1^·Å^−2^ on all atoms in each system and executed 100 ps. In order to balance the solvent density, a short equilibration simulation was carried out for 50 ps, and all atoms were constrained by 5 kcal·mol^−1^·Å^−2^ under 1 atm pressure in the isothermal isobaric (NPT) ensemble. Then, we performed 1.5 ns equilibration in the NPT ensemble for each system. The first 1.0 ns was divided into five stages, including a restriction force of 5.0 kcal·mol^−1^·Å^−2^, 4.0 kcal·mol^−1^·Å^−2^, 3.0 kcal·mol^−1^·Å^−2^, 2.0 kcal·mol^−1^·Å^−2^, and 1.0 kcal·mol^−1^·Å^−2^ for each system, respectively, and carried out for 200 ps in each stage. After that, a 500 ps equilibration for each system without imposing the limiting force was performed. Finally, we used the PMEMD program in AMBER14 to carry out a 100 ns production of MD simulations at 310.0 K with 1 atm in the NPT ensemble without any restraint for each system. In the simulation process, the SHAKE algorithm [61]. was used to bound hydrogen bond length and the particle-mesh Ewald (PME) [62]. method was used to deal with long-range Coulomb interactions. The non-bonded cutoff value was set as 10.0 Å to deal with non-bonded interactions, and periodic boundary conditions were applied to avoid edge effects. The time step size was set as 2 fs. We kept a record of coordinate trajectory every 2 ps for all the production trajectories.

### 3.7. Binding Free Energy Calculations

In this study, we used the MM-GBSA [63,64,65,66]. method to calculate the binding free energy of each system. In this process, an average of 2000 structures were extracted at an interval of 10 ps from the last 20 ns of MD trajectory. We used the equations below to calculate the binding free energy:

$$\begin{array}{c} \Delta G_{\mathrm{bind}}=\Delta G_{\mathrm{complex}}-\Delta G_{\mathrm{receptor}}-\Delta G_{\mathrm{ligand}}\\ \Delta G_{\mathrm{bind}}=\Delta H-T\Delta S\approx \Delta E_{\mathrm{MM}}+\Delta G_{\mathrm{solv}}-T\Delta S\\ \Delta E_{\mathrm{MM}}=\Delta E_{\mathrm{internal}}+\Delta E_{\mathrm{ele}}+\Delta E_{\mathrm{vdW}}\\ \Delta G_{\mathrm{solv}}=\Delta G_{\mathrm{GB}}+\Delta G_{\mathrm{NP}} \end{array}$$

The binding free energy (∆G_bind_) is the sum of the enthalpy term (∆H) and entropy term (−T∆S). ∆H of the system is the summation of the interaction energy of the gas phase between the protein–ligand (∆E_MM_) and the solvated free energy (∆G_solv_). ∆E_MM_ is obtained by adding the internal energy (∆E_internal_, consists of the energies of bonds, angels, and torsions), the electrostatic interaction energy (∆E_ele_), and the van der Waals interaction energy (∆E_vdW_). ∆G_solv_ is the sum of the polar solvation free energy (∆G_GB_) and the nonpolar solvation free energy (∆G_NP_).

### 3.8. Per-Residue Free Energy Decomposition Analysis

We decomposed per-residue free energy decomposition using the 2000 structures collected from the last 20 ns of MD trajectory at an interval of 10 ps. The MM-GBSA method was employed to calculate per-residue free energy decomposition (∆G_MM-GBSA_) with the following equation:

$$\Delta G_{\mathrm{MM}-\mathrm{GBSA}}=\Delta E_{\mathrm{vdW}}+\Delta E_{\mathrm{ele}}+\Delta E_{P}+\Delta E_{\mathrm{NP}}$$

In this formula, ∆E_vdW_ represents the van der Waals interaction energy, ∆E_ele_ represents the electrostatic interaction energy, ∆E_P_ represents the polar solvation free energy, and ∆E_NP_ represents the nonpolar solvation free energy.

## 4. Conclusions

In summary, we first downloaded natural products from the TargetMol, ChemDiv, and InterBioScreen databases (70,902 molecules in total). Then, we implemented docking study to virtually screen chemicals that could bind to ACE2. Then, MM-GBSA ΔG values and ADME properties were calculated and predicted for 500 top-ranked ligands. Subsequently, we selected and clustered 298 molecules into 12 categories. As a result, four natural compounds with strong binding affinity activities to ACE2 and known medicinal effects were selected as potential ACE2 inhibitors to prevent the invasion of SARS-CoV-2. Afterwards, we performed MD simulations on these four systems and found that the compound STOCK1N−53429 could not bind with ACE2 stably, and the compound 132-98-9 also showed changes in the binding site during the MD simulation. The results showed that the compounds 154-23-4 and STOCK1N-07141 are the most promising candidates deserving further research.

## Figures and Tables

**Table 1:** The cluster results and the compound number contained in each cluster.

categories	1	2	3	4	5	6	7	8	9	10	11	12
numbers	26	3	6	1	3	10	112	12	1	2	121	1

**Table 2:** The structures and ADME properties of the 12 top ligands.

Molecule	Structures	^a^ mol MW	^b^ QPlogPo/w	^c^ Donor HB	^d^ Accept HB	^e^ PSA	^f^ %Human Oral Absorption
154-23-4	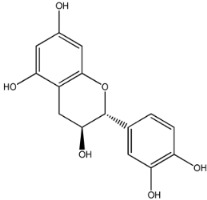	290.272	0.459	5	5.450	114.862	60.524
STOCK1N-25862	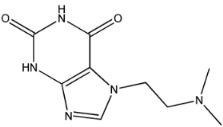	223.234	−0.792	2.000	7.000	105.840	52.040
STOCK1N-20317	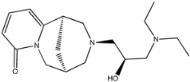	319.442	0.732	1.000	8.700	59.123	66.608
132-98-9	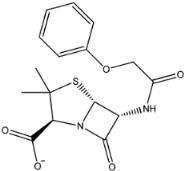	350.389	2.076	1.250	7.000	116.295	62.569
STOCK1N-81825	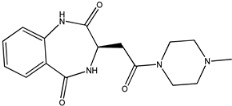	316.359	−0.373	1.000	9.000	109.685	52.306
STOCK1N-79835	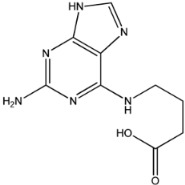	236.233	−0.301	5.000	6.500	139.745	40.010
STOCK1N−53429	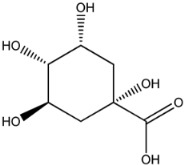	192.168	−1.256	5.000	7.850	127.861	38.810
STOCK1N-07141	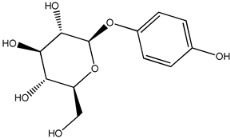	272.254	−0.976	5.000	10.000	120.231	56.911
STOCK1N-05528	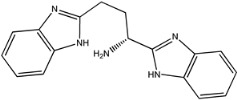	291.355	2.229	4.000	4.000	79.958	80.263
STOCK1N-20017	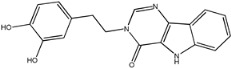	321.335	2.216	3.000	5.500	96.236	84.644
STOCK1N-74592	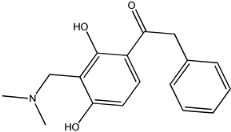	285.342	3.083	1.000	4.500	69.235	86.381
STOCK1N-88912	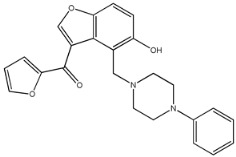	402.449	3.630	1.000	6.750	65.991	100.000

^a^ MW ≤ 500; ^b^ QPlogPo/w ≤ 5; ^c^ Donor HB ≤ 5; ^d^ Accept HB ≤ 10; ^e^ PSA ≤ 140Å^2^; and ^f^ Percent Human Oral Absorption > 25.

**Table 3:** The results of docking and interaction residues between the top 12 ligands and ACE2.

Cluster No.	Compound ID	Docking Interaction	Interacting Residues	Glide Score	Docking Score	ΔG (kcal/mol)
1	154-23-4	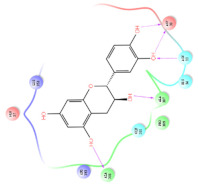	Asp30, Asn33, Ala386, Ala387	−5.418	−5.148	−37.592
2	STOCK1N-25862	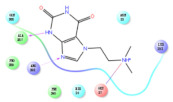	Glu37, Ala387, Arg393	−4.677	−4.584	−22.063
3	STOCK1N-20317	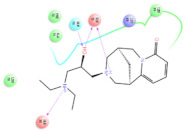	Glu35, Glu75, Gln76	−5.156	−4.905	−42.895
4	132-98-9	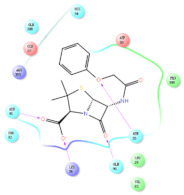	Lys26, Asn33, Asn90, Gln96	−4.335	−4.335	−18.899
5	STOCK1N-81825	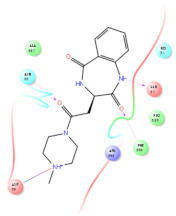	Asp30, Asn33, Glu37, Phe390,	−4.839	−4.813	−33.580
6	STOCK1N-79835	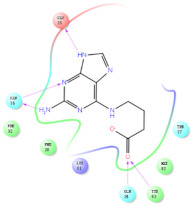	Gln24, Glu35, Gln76, Tyr83	−4.959	−4.947	−22.213
7	STOCK1N−53429	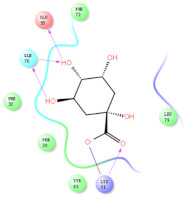	Lys31, Glu35, Gln76,	−5.923	−5.923	−19.312
8	STOCK1N-07141	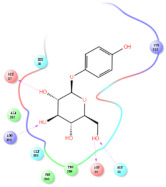	Asp30, Asn33, Glu37, Arg393	−4.898	−4.898	−27.518
9	STOCK1N-05528	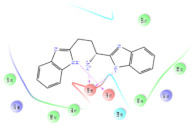	Glu35, Glu75	−5.002	−4.855	−41.649
10	STOCK1N-20017	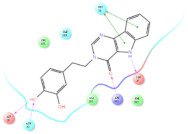	Asp30, His34, Asn33, Glu37, Arg393	−4.543	−4.542	−37.204
11	STOCK1N-74592	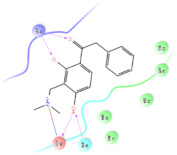	Lys31, Glu35, Gln76	−6.193	−5.881	−33.179
12	STOCK1N-88912	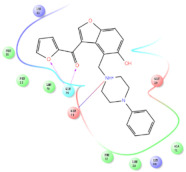	Gln75, Glu76	−4.134	−4.094	−32.160

**Table 4:** Binding free energy (kcal/mol) of each system, and the energy contribution of each component.

Contribution (kcal/mol)	Complexes		
	132-98-9/ACE2	154-23-4/ACE2	STOCK1N-07141/ACE2
∆E_ele_	173.35(23.03)	−38.55(11.61)	−48.92(20.00)
∆E_vdW_	−15.22(7.94)	−17.99(3.39)	−14.62(4.28)
∆G_GB_	−166.61(21.36)	49.47(10.29)	54.00(15.97)
∆G_SA_	−2.12(1.09)	−3.29(0.39)	−2.92(0.49)
∆E_gas_	158.12(22.65)	−56.54(11.58)	−63.54(19.00)
∆E_solv_	−168.73(21.46)	46.18(10.12)	51.08(15.66)
∆G_bind_	−10.61(6.97)	−10.36(3.61)	−12.46(5.52)

**Table 5:** Hydrogen bond distribution for each system.

Complex	Acceptor	DonorH	Donor	Frac
132-98-9/ACE2	MOL@O1	TYR_83@HH	TYR_83@OH	0.1460
154-23-4/ACE2	MOL@O2	ASN_90@HD21	ASN_90@ND2	0.3216
	GLN_388@OE1	MOL@H13	MOL@O5	0.3006
STOCK1N-07141/ACE2	GLU_37@OE22	MOL@H6	MOL@O3	0.5430
	GLU_37@OE2	MOL@H7	MOL@O4	0.5300
	ALA_387@O	MOL@H15	MOL@O6	0.5072
	GLU_37@OE1	MOL@H7	MOL@O4	0.4849
	GLU_37@OE1	MOL@H6	MOL@O3	0.4359
	MOL@O4	HIP_34@HD1	HIP_34@ND1	0.3574

## Data Availability

Data sharing is not applicable to this article.
